# Principal component analysis exploring the association between antibiotic resistance and heavy metal tolerance of plasmid-bearing sewage wastewater bacteria of clinical relevance

**DOI:** 10.1099/acmi.0.000095

**Published:** 2020-02-10

**Authors:** Manisha Mandal, Saumendra Nath Das, Shyamapada Mandal

**Affiliations:** ^1^​ Department of Physiology, MGM Medical College and LSK Hospital, Kishanganj-855107, India; ^2^​ Department of Zoology, University of Gour Banga, Malda-732103, India

**Keywords:** sewage wastewater bacteria, multiple-antibiotic resistance phenotypes, heavy metal tolerance, plasmid, co-resistance, 16s RNA gene sequence

## Abstract

This paper unravels the occurrence of plasmid-mediated antibiotic resistance in association with tolerance to heavy metals among clinically relevant bacteria isolated from sewage wastewater. The bacteria isolated were identified following conventional phenotypic and/or molecular methods, and were subjected to multiple-antibiotic resistance (MAR) profiling. The isolates were tested against the heavy metals Hg^2+^, Cd^2+^, Cr^2+^ and Cu^2+^. SDS-PAGE and agarose gel electrophoretic analyses were performed, respectively, for the characterization of heavy metal stress protein and R-plasmid among the isolated bacteria. Principal component analysis was applied in determining bacterial resistance to antibiotics and heavy metals. Both lactose-fermenting (* Escherichia coli *) and non-fermenting (* Acinetobacter baumannii* and *Pseudomonas putida *) Gram-negative bacterial strains were procured, and showed MAR phenotypes with respect to three or more antibiotics, along with resistance to the heavy metals Hg^2+^, Cd^2+^, Cr^2+^ and Cu^2+^. The Gram-positive bacteria, *Enterococcus faecalis *, isolated had ‘ampicillin–kanamycin–nalidixic acid’ resistance. The bacterial isolates had MAR indices of 0.3–0.9, indicating their (* E. faecalis *, *E. coli *, *A. baumannii* and *P. putida *) origin from niches with high antibiotic pollution and human faecal contamination. The Gram-negative bacteria isolated contained a single plasmid (≈54 kb) conferring multiple antibiotic resistance, which was linked to heavy metal tolerance; the SDS-PAGE analysis demonstrated the expression of heavy metal stress proteins (≈59 and ≈10 kDa) in wastewater bacteria with a Cd^2+^ stressor. The study results grant an insight into the co-occurrence of antibiotic resistance and heavy metal tolerance among clinically relevant bacteria in sewage wastewater, prompting an intense health impact over antibiotic usage.

## Introduction

The rampant, haphazard and injudicious use of affordable and easily available antibiotics (from the market even without prescription) is leading to the emergence of clinically relevant multidrug-resistant (MDR) bacteria [1] in various ecological niches, including water bodies such as sewage systems that act as mixing vessels for hospital, agricultural and domestic effluents. Metal ions also aid the development and dissemination of antibiotic-resistant potential bacterial pathogens in environmental as well as clinical settings, and sustain the phenotypic expression associated with heavy metal–antibiotic resistance co-selection [2, 3]. As reported earlier [4, 5], hospital wastewater has been recognized as an antibiotic resistance hotspot in receiving a mixture of antibiotics and other pharmaceutical agents, such as disinfectants, and patient excretory materials containing MDR bacterial pathogens. Therefore, the biological components, mostly the bacterial populations, are exposed to chemical substances such as antibiotics and heavy metal salts in the sewage and acquire resistance to antibiotics as well as heavy metals, intrinsically (through chromosomal gene mutation) and/or extrinsically (through the acquisition of R-plasmid) [6]. Plasmid-mediated antibiotic resistance in association with heavy metal tolerance among environmental and clinical bacteria has been reported previously [7, 8].

Antibiotic resistance is growing among diverse bacterial (pathogenic) populations and is a huge clinical problem that complicates the medical use of antibiotics against several MDR infections with Gram-negative pathogens – *Escherichia coli *, *Acinetobacter baumannii *, *Pseudomonas aeruginosa* and *Klebsiella pneumoniae* – as well as Gram-positive *Staphylococcus aureus* [9]. However, the MDR Gram-negative pathogenic bacteria, which are linked to arrays of pathologies and show resistance even to newer antibiotics, are emerging and spreading worldwide at a fast pace [10]. The bacterial pathogens have different multiple-antibiotic resistance (MAR) phenotypes with three or more antibiotics, and MAR indices vary greatly too [6, 7].

Bacterial antibiotic resistance has not been reported solely in clinical settings; MDR bacteria are also found in agricultural settings as well as in environments such as soil and water (fresh water as well as sewage wastewater) [6, 9]. Acquaintance with the local status of antibiotic resistance among potentially pathogenic bacteria in different settings is, thus, crucial in combating the phenomenon [7, 8]. Therefore, the current study was undertaken to ascertain the association between antibiotic resistance and heavy metal tolerance among plasmid-bearing potentially pathogenic bacteria isolated from the sewage system surrounding the University of Gour Banga campus, Malda, India.

## Methods

### Bacteriological profiles of sewage wastewater

The sewage samples, in the form of wastewater (*n*=6), were collected from the sewage system surrounding the campus of the University of Gour Banga, Malda, India, in screw-capped sterilized sampling bottles (Hi-Media, India), and transported to the Laboratory of Microbiology and Experimental Medicine, Department of Zoology, University of Gour Banga, India.

The bacteriological analyses of sewage wastewater samples followed a previously described method [6, 7]. For bacterial growth enrichment, the samples (100 µl each) were incubated in nutrient broth (Hi-Media, India) for 24 h at 35 °C, and thereafter, following the streak plate dilution technique, morphologically distinct colonies of the isolated bacteria were procured using different selective media (MacConkey agar and brilliant green bile agar for *E. coli *, and cetrimide agar, for *Pseudomonas* spp.), as well as general purpose media, such as nutrient agar for *Enterococcus* spp. [6]. Further subcultures were performed in order to obtain pure cultures of bacteria from each of the collected sewage samples. The bacteria isolated (*n*=6; strain code: ST1, ST2, ST3, ST4, ST5 and ST6) were characterized and identified following conventional phenotypic methods as per the standard protocol of Halt [11] and Forbes *et al*. [12].

### Molecular characterization of bacterial isolates

Two sewage water bacterial strains – ST1 (LMEM305) and ST5 (LMEM306) – were subjected to 16S rRNA gene sequencing and phylogenetic analysis for identity confirmation, and for this purpose the pure bacteria cultures were sent to Eurofins Genomics India Private Limited (Karanataka, India). The ≈1.5 kb 16S rRNA gene fragment, from the extracted test bacterial genomic DNA, was PCR-amplified, and the products were sequenced using a universal primer: 27F: 5′-AGAGTTTGATCMTGGCTCAG-3′ and 1492R: 5′-CGGTTACCTTGTTACGACTT-3′. The sequenced data (partial 16S rRNA gene sequences) were aligned by using the National Center for Biotechnology Information’s (NCBI’s) GenBank, in order to determine the closest known relatives of the sequence obtained, by using similarity searches (for nucleotide homology) through blast analysis (blastn) [13], and a phylogenetic tree was constructed using the neighbour-joining method. The 16S rRNA gene sequences of LMEM305 and LMEM306 strains have been deposited with the NCBI GenBank accession numbers MK182778 and MK182775, respectively.

### Antibiotic susceptibility testing

The antibiotic susceptibility patterns of the isolated bacteria were determined following disc diffusion [14], against 10 antibiotics (Mueller–Hinton agar; Hi-Media, India): ampicillin (Am; 10 µg disc^−1^), chloramphenicol (Cm; 30 µg disc^−1^), cefotaxime (Cf; 30 µg disc^−1^), cefoxitin (Cx; 30 µg disc^−1^), gentamicin (Gm; 10 µg disc^−1^), imipenem (Im; 10 µg disc^−1^), kanamycin (Km; 30 µg disc^−1^), nalidixic acid (Nx; 30 µg disc^−1^), piperacillin (Pc; 100 µg disc^−1^) and tetracycline (Tc; 30 µg disc^−1^). Incubation was performed at 37 °C, for 24 h, and the results, in terms of zone diameter of inhibition (ZDI; nearest whole in millimetres) from the test antibiotic action, were interpreted according to the Clinical and Laboratory Standards Institute (CLSI) criteria [15]. The MAR indices for the isolated bacteria were calculated using the formula stated earlier [16], and MAR phenotype profiles were generated for the isolates with resistance to three or more test antibiotics [17]. MAR indices of >0.2 indicated that the bacteria originated from niches with a high risk of antibiotic pollution [18], while MAR indices of >0.4 indicated that the bacterial originated from niches with human faecal contamination [19, 20].

### Determination of bacterial heavy metal tolerance

The maximum tolerance-concentration (MTC) values of four heavy metals – HgCl_2_ (Hg^2+^), CdCl_2_ (Cd^2+^), K_2_Cr_2_O_7_ (Cr^6+^) and CuSo_4_ (Cu^2+^) – were determined for the isolated sewage wastewater bacteria by agar dilution method as described earlier, utilizing inoculum of ≈10^4^ c.f.u. spot^−1^ [6]. The heavy metal concentrations used for the study were: Hg^2+^ (3–50 μg ml^−1^), Cd^2+^ (25–1000 μg ml^−1^), Cr^2+^ (25–500 μg ml^−1^) and Cu^2+^ (200–1000 μg ml^−1^). The sewage wastewater bacteria exhibiting growth in the presence of ≥3 μg ml^−1^ of the test heavy metals were categorized as heavy-metal-tolerant.

### Extraction of bacterial heavy metal stress protein and SDS-PAGE analysis

Bacterial protein was extracted following the method described by Ismail *et al*. [21], with modifications. Subcultures of the test bacterial strains were performed in order to prepare 25 ml nutrient broth culture, after incubation for 48 h at 37 °C, and thereafter, cell-free supernatant (CFS) was obtained by centrifugation (10 000 r.p.m. for 10 min at 4 °C) and syringe filtration. The CFS was treated with ammonium sulphate (60%) at 4 °C for 24 h for protein precipitation, and then centrifuged (12 000 r.p.m. for 15 min at 4 °C), extracted and washed with sterilized double-distilled water. Finally, the pellet of protein was mixed with phosphate buffer solution (1 ml, pH 7.2) in combination with 0.6 % SDS and stored at 4 °C for further use.

The extracted protein from the test bacterial strains was subjected to molecular weight approximation by glycine SDS-PAGE (sodium dodecyl sulfate-polyacrylamide gel electrophoresis) analysis [22] using a vertical slab gel apparatus (Tarsons, India) with 4 % stacking and 15 % separating gels, and High-Range Protein Molecular Weight Markers (Hi-Media, India). Following electrophoresis for 5 h at 70 V, the gel was stained with Coomassie brilliant blue (Hi-Media, India) and then destained in 30 % (v/v) methanol/10 % (v/v) glacial acetic acid in order to visualize the protein bands. The molecular weight of the bacterial proteins in the gel was calculated from the relative mobility of the bands from mid-range protein molecular weight markers (SRL, India): *y*=20.72 x+98.62 with *R* ^2^=0.984.

### Bacterial plasmid profiles

As mentioned earlier [6], the plasmid DNA from the Gram-negative test bacteria (resistant to three or more antibiotics and heavy metals) were isolated following the protocol of Kado and Liu [23]: briefly, from young plate cultures on nutrient agar, the test bacterial colonies were scraped into an Eppendorf tube containing lysing buffer, mixed with phenol/chloroform and boiled in a water bath for 60 min at 55 °C. The upper aqueous phase, after centrifugation for 10 min at 5000 r.p.m. (4 °C), was treated with cold isopropanol, and the precipitated plasmid (on centrifugation) was dissolved in tris-EDTA (TE) buffer.

Agarose gel electrophoresis was carried out on the isolated plasmids, following the method described by Maniatis *et al*. [24], in a tris-borate buffer system with horizontal electrophoresis apparatus (GeNei, India), using a 0.8 % agarose gel slab, for 3 h at 50 V. The plasmid DNA bands in the gel after ethidium bromide staining were visualized and documented using the Gel Doc system.

In order to investigate the loss of plasmid, randomly selected sewage wastewater bacteria (strains ST1, ST4 and ST6) were subjected to plasmid curing with sodium dodecyl sulfate (SDS), following the protocol of Anjanappa *et al*. [25], as described elsewhere [6, 26]. Briefly, the bacterial strains were grown in nutrient broth (Hi-Media, India) supplemented with 100 µg ml^−1^ of SDS for 7 days at 37 °C; thereafter, the broth cultures were streaked on nutrient agar plates, and 25 colonies (for each test bacterium) were picked, following 24 h incubation at 37 °C. The loss of antibiotic resistance and heavy metal tolerance (among the selected colonies), along with the loss of plasmid, was determined based on the resistance patterns of the cured bacterial strains and the absence of plasmid in the gel following electrophoresis for the cured bacterial strains.

### Principal component analysis

Multivariate statistical techniques, such as principal component analysis (PCA), were applied (using Excel Stat 2019 version 2019.3.2) to assess the association between pair-wise variables (bacterial antibiotic resistance and heavy metal tolerance). The number of principal components and the factors were selected according to the Kaiser criterion [27], and the factors displaying eigenvalues >1.00 were accounted [28]. The statistical significance of differences was evaluated using one-way analysis of variance (using Excel 2010; Microsoft Office 2010, Microsoft Corp., USA) with *P*-values <0.05.

## Results

### Sewage wastewater bacterial isolates

**Fig. 1.**
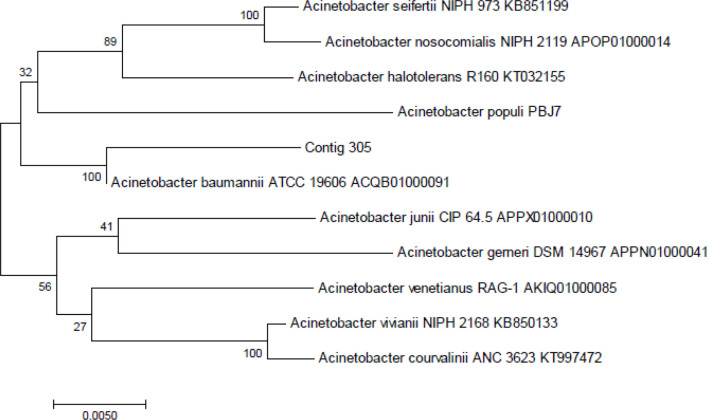
16S rRNA gene sequence-based phylogenetic tree for *A. baumannii* strain LMEM 305 (contig 305) compared with the sequences of closely related reference bacterial strains retrieved from the NCBI’s GenBank database. Digits shown at the nodes represent the bootstrap values (for a total of 1000 replicates).

**Fig. 2.**
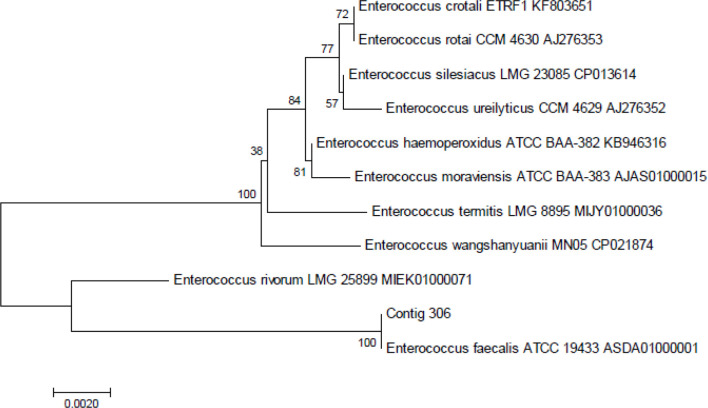
16S rRNA gene sequence-based phylogenetic tree for *E. faecalis* strain LMEM 306 (contig 306) compared with the sequences of closely related reference bacterial strains retrieved from the NCBI’s GenBank database. Digits shown at the nodes represent the bootstrap values (for a total of 1000 replicates).

All the sewage-water samples showed bacterial contamination and a total of six bacterial strains were isolated, one from each of the samples, of which five were Gram-negative rods (strain code: ST1, ST2, ST3, ST4 and ST6) and one was a Gram-positive coccus (strain code: ST5). The Gram-negative lactose-fermenting tryptophanase and catalase-positive strains (*n*=3; strain code: ST2, ST3 and ST4) with no gelatinase property were all designated as *E. coli *, while the Gram-positive lactose-fermenting coccus (*n*=1; strain code: ST5) showing citrate-positive but indole- and catalase-negative test results was designated as *Enterococcus faecalis *. Among the lactose-non-fermenting indole- and nitrate-negative catalase-producing Gram-negative rods, the strain (code: ST6) with oxidase and gelatinase properties was identified as *Pseudomonas putida *, while the arabinose-utilizing strain (code: ST1) was identified as *A. baumannii *. Further, based on the 16S rRNA gene sequences, phylogenetic trees were constructed to recognize the genetic relationship among the bacterial strains (Figs 1 and 2). The LMEM 305 strain (isolation strain code: ST1) showed closest similarity to the *A. baumannii* ATCC 19606 ACQB01000091 strain, while the LMEM 306 strain (isolation strain code: ST5) expressed closest similarity to the *E. faecalis* ATCC 19433 ASDA 01000001 strain, and, therefore, their identities were confirmed, respectively, as *A. baumannii* LMEM 305 (NCBI GenBank accession numbers MK182778) (Fig. 1) and *E. faecalis* LMEM 306 (NCBI GenBank accession numbers MK182775) (Fig. 2).

### Plasmid-mediated antibiotic resistance and heavy metal tolerance of isolated bacteria

The disc diffusion susceptibility test results (in terms of ZDI) for the isolated bacteria are shown in Table 1. The antibiotic resistance phenotypes and MAR indices for the sewage wastewater bacteria are shown in Table 2. All of the Gram-negative isolates showed MAR phenotypes with respect to three or more agents, and among them the lactose-fermenting strains were eight-drug resistant, while the non-fermenters showed resistance to four antibiotics; the Gram-positive *E. faecalis* strain had a three-drug (Am–Km–Nx) resistance phenotype. The MAR indices, which ranged from 0.3 to 0.9 for the clinically relevant sewage wastewater bacteria, are depicted in Table 2.

**Table 1.** The ZDI for sewage wastewater bacterial isolates (*n*=6)

Antibiotic	ZDI (mm)					
	* A. baumannii *	* E. coli *			* E. faecalis *	* P. putida *
	ST1	ST2	ST3	ST4	ST5	ST6
Am	6	6	6	6	6	6
Cm	10	28	27	28	30	10
Cf	13	6	6	6	15	19
Cx	6	6	6	6	20	6
Gm	22	9	10	10	23	25
Ip	14	6	12	12	18	22
Km	19	6	6	6	6	25
Nx	17	6	6	6	6	8
Pc	16	6	6	6	25	20
Tc	19	8	8	9	25	14

Am, ampicillin; Cm, chloramphenicol; Cf, cefotaxime; Cx, cefoxitin; Gm, gentamicin; Ip, imipenen; Km, kanamycin; Nx, nalidixic acid; Pc, piperacillin; Tc, tetracycline.

**Table 2.** The antibiotic resistance phenotypes and MAR indices of sewage wastewater bacteria (*n*=6)

Strain	Strain identity	Antibiotic resistance phenotype	MAR index
ST1	* A. baumannii *	Am–Cx–Ip–Pc	0.4
ST2	* E. coli *	Am–Cx–Cf–Gm–Ip–Km–Nx–Pc–Tc	0.9
ST3	* E. coli *	Am–Cx–Cf–Gm–Ip–Km–Nx–Pc–Tc	0.9
ST4	* E. coli *	Am–Cx–Cf–Gm–Ip–Km–Nx–Pc–Tc	0.9
ST5	* E. faecalis *	Am–Km–Nx	0.3
ST6	* P. putida *	Am–Cx–Nx	0.3

MAR: multiple antibiotic resistance index; antibiotic abbreviations are shown in Table 1.

**Fig. 3.**
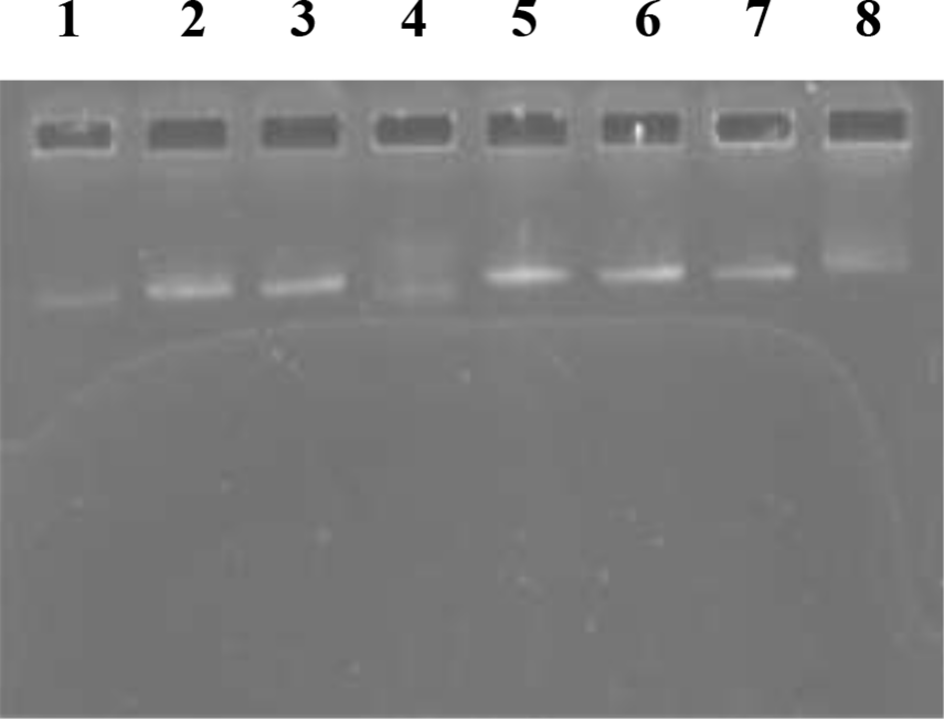
Plasmid profile of sewage wastewater bacterial isolates: lane 1, *E. coli* ST2; lane 2, *E. coli* ST3; lane 3, *E. coli* ST4; lane 4, *E. coli* ST4 (cured strain); lane 5, *E. coli* V517 (≈54 kb) standard molecular marker; lane 6, *A. baumannii* ST1; lane 7, *P. putida* ST6; lane 8, *P. putida* ST6 (cured strain). Note the presence of chromosomal DNA smears and the absence of plasmid DNA bands in cured bacterial strains (lanes 4 and 8).

The levels of heavy metal tolerance for the sewage wastewater bacteria are depicted in Table 3, while the curing of antibiotic resistance and heavy metal tolerance in test bacteria is shown in Table 4. Among the *E. coli* isolates, *E. coli* ST4 originally showed resistance to Hg^+2^, Cd^+2^, Cr^+6^ and Cu^+2^, along with MAR to nine antibiotics, and the strain lost Am–Cx–Cf–Tc resistance, and also became sensitive to all the heavy metals tested, while *A. baumannii* ST1 (antibiotic resistance pattern: Am–Cx–Ip–Pc) and *P. putida* ST6 (antibiotic resistance pattern: Am–Cx–Nx) showed resistance to three heavy metals (Cd^+2^, Cr^+6^ and Cu^+2^) and lost resistance to Am–Cx–Cd^+2^–Cr^+6^–Cu^+2^ on SDS treatment. In the current study, the concomitant loss of a bacterial plasmid (≈54 kb) occurred, along with the loss of heavy metal tolerance and antibiotic resistance properties. The plasmid electropherotypes for the sewage wastewater bacteria (* A. baumannii* ST1, *E. coli* ST4 and *P. putida* ST6) are represented in Fig. 3.

**Table 3.** Heavy metal tolerance level for sewage wastewater bacterial isolates (*n*=6)

Bacterial strain	Heavy metal tolerance (µg ml^−1^)			
	Hg^2+^	Cd^2+^	Cr^6+^	Cu^2+^
* A. baumannii* ST1	2	25	50	650
* E. coli* ST2	2	100	75	650
* E. coli* ST3	2	100	75	650
* E. coli* ST4	3	100	75	650
* E. faecalis* ST5	2	50	250	750
* P. putida* ST6	2	250	300	850

**Table 4.** Antibiotic resistance and heavy metal tolerance patterns of sewage wastewater bacterial isolates and their cured derivatives

Bacterial Strain	Original strain			Cured strain			
	Resistance pattern			Sensitivity to		Resistance retained	
	Antibiotic	HM	Antibiotic		HM	Antibiotic	HM
* E. coli* ST4	Am–Cx–Cf–Gm–Ip–Km–Nx–Pc–Tc	Hg^+2^,Cd^+2^, Cr^+6^,Cu^+2^	Am-Cx-Cf-Tc		Hg^+2^,Cd^+2^, Cr^+6^,Cu^+2^	Gm–Ip–Km–Nx–Pc	–
* A. baumannii* ST1	Am–Cx–Ip–Pc	Cd^+2^, Cr^+6^,Cu^+2^	Am-Cx		Cd^+2^, Cr^+6^, Cu^+2^	Ip–Pc	–
* P. putida* ST6	Am–Cx–Nx	Cd^+2^, Cr^+6^,Cu^+2^	Am-Cx		Cd^+2^, Cr^+6^, Cu^+2^	Nx	–

HM: heavy metal; the antibiotic abbreviations are shown in Table 1.

### SDS-PAGE analysis of bacterial heavy metal stress protein

**Fig. 4.**
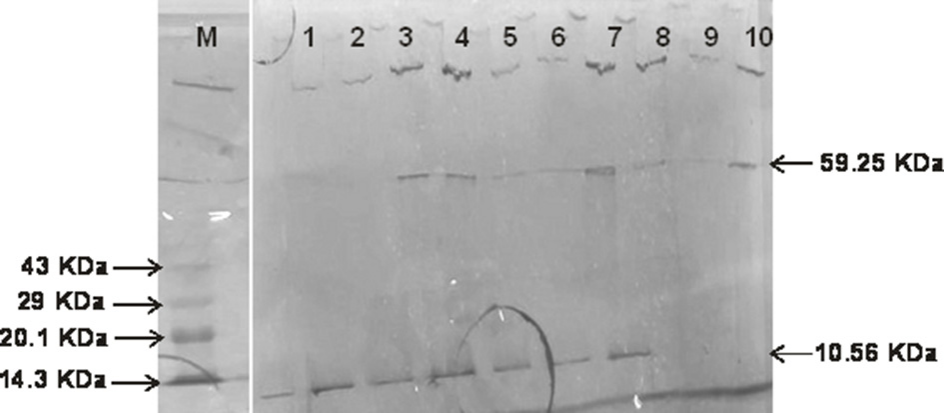
SDS-PAGE profiles of protein isolated from sewage wastewater bacterial isolates: M, mid range protein molecular marker; lane 1, *A. baumannii* ST1 (untreated control); lane 2, *A. baumannii* ST1 (Cd^+2^-treated); lane 3, *E. coli* ST2 (Cd^+2^-treated); lane 4, *E. coli* ST3 (Cd^+2^-treated); lane 5, *E. coli* ST4 (Cd^+2^-treated); lane 6, *E. faecalis* ST5 (Cd^+2^-treated); lane 7, *P. putida* ST6 (Cd^+2^-treated); lane 8, *E. coli* ST3 (untreated control); lane 9, *E. faecalis* ST5 (untreated control); lane 10, *P. putida* ST6 (untreated control).

SDS-PAGE analysis of heavy metal stress (Cd: 25 µg ml^−1^) protein among sewage wastewater bacteria revealed the expression of single to double protein bands of two different sizes: 59.25 and 10.56 KDa (Fig. 4).

### Principal component analysis

**Fig. 5.**
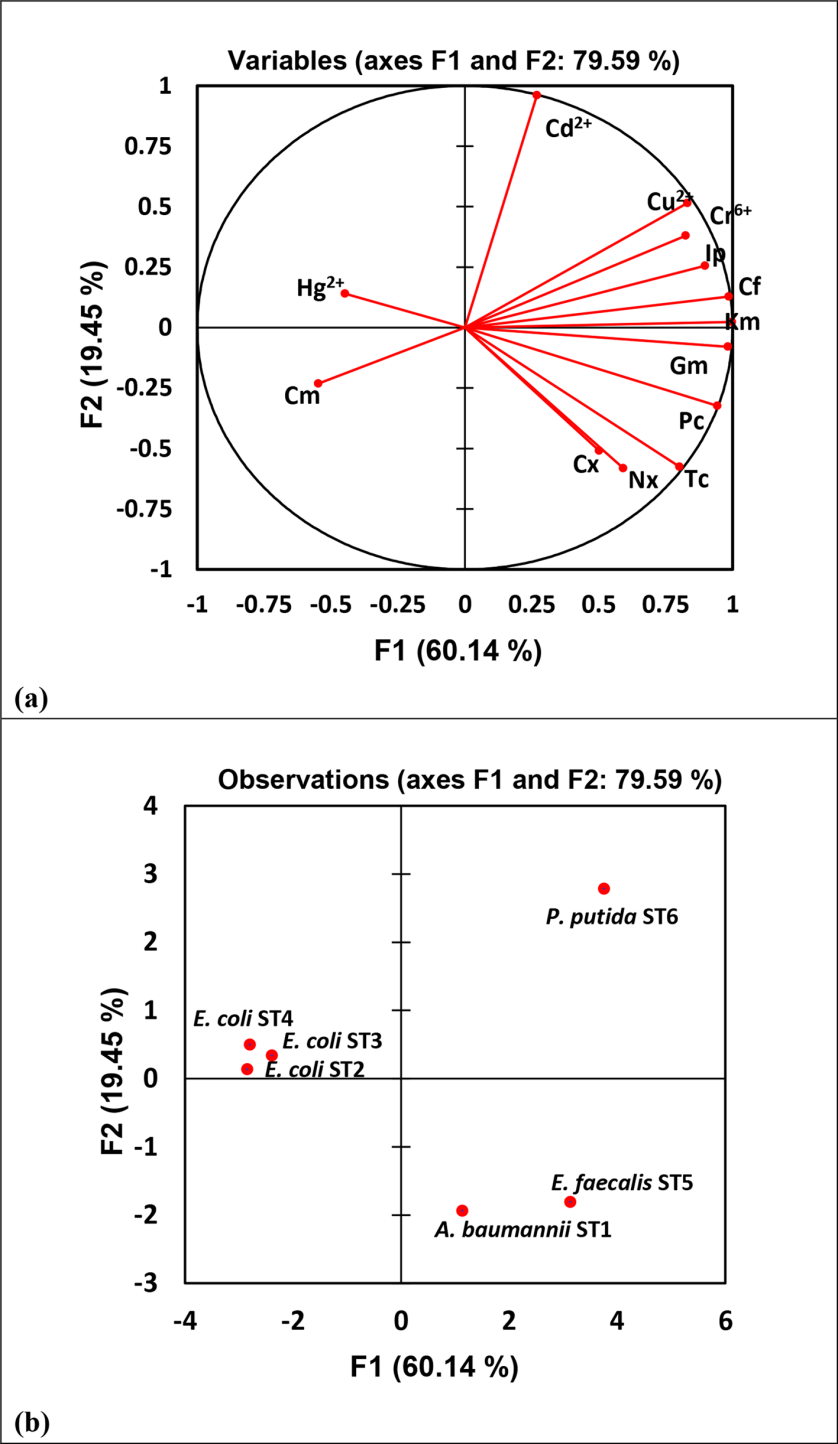
PCA of antibiotic resistance and heavy metal tolerance among sewage wastewater bacteria: (a) variable projection of F1 and F2 factors (antibiotics and heavy metals); (b) projection of six sewage bacterial isolates in F1 and F2 factors.

**Fig. 6.**
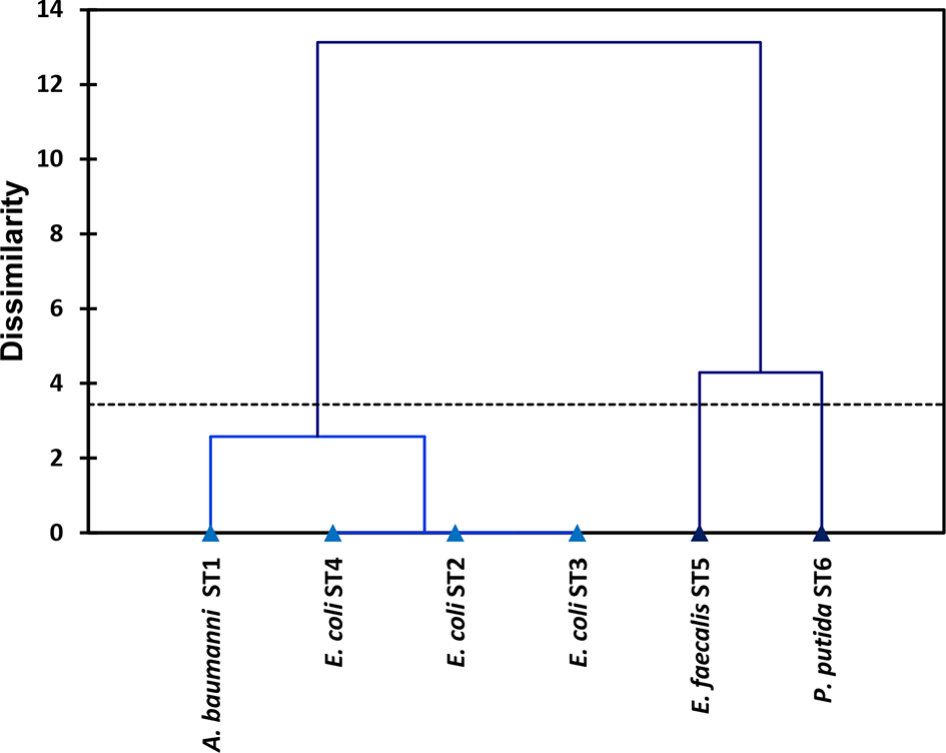
Hierarchical clustering analysis aligning the isolated sewage wastewater bacterial strains (*n*=6) on the basis of their antibiotic resistance and heavy metal tolerance profiles.

The nature and direction of correlation between bacterial antibiotic resistance and heavy metal tolerance are represented in Table 5. The association between the factors and the test variables (antibiotic–heavy metal resistance) was evident from the projection of the resistance properties onto the plane formed by the F1 and F2 factors, as depicted in PCA (Fig. 5), and also from the Pearson correlation matrix (Table 5). The foremost importance of grouping the bacteria based upon their properties of resistance to antibiotics and tolerance to heavy metals was accomplished by analysing the hierarchical clustering, as represented in Fig. 6.

**Table 5.** Correlation matrix (Pearson’s correlation coefficients) among bacterial antibiotic resistance and heavy metal tolerance

Variables	Cm	Cf	Cx	Gm	Ip	Km	Nx	Pc	Tc	Hg^2+^	Cd^2+^	Cr^6+^	Cu^2+^
**Cm**	**1**												
**Cf**	−0.647	1											
**Cx**	0.405	0.362	1										
**Gm**	−0.649	0.974*	0.421	1									
**Ip**	−0.513	0.901*	0.355	0.864*	1								
**Km**	−0.538	0.988*	0.502	0.977*	0.892*	1							
**Nx**	−0.636	0.564	0.206	0.720	0.366	0.569	1						
**Pc**	−0.400	0.885*	0.676	0.946*	0.764	0.937*	0.720	1					
**Tc**	−0.205	0.707	0.785	0.818*	0.593	0.792	0.746	0.954*	1				
**Hg^2+^**	0.302	−0.420	−0.200	−0.421	−0.178	−0.425	−0.356	−0.430	−0.340	1			
**Cd^2+^**	−0.352	0.388	−0.340	0.182	0.464	0.293	−0.414	−0.056	−0.338	−0.026	1		
**Cr^6+^**	−0.207	0.825*	0.510	0.713	0.839*	0.843*	0.037	0.679	0.494	−0.283	0.599	1	
**Cu^2+^**	−0.416	0.869*	0.293	0.743	0.867*	0.848*	0.082	0.629	0.394	−0.293	0.727	0.967*	1

The antibiotic abbreviations are shown in Table 1.

*Values (Pearson’s *R*) are different from ‘zero’ with a significance level of *P*<0.05.

**Fig. 7.**
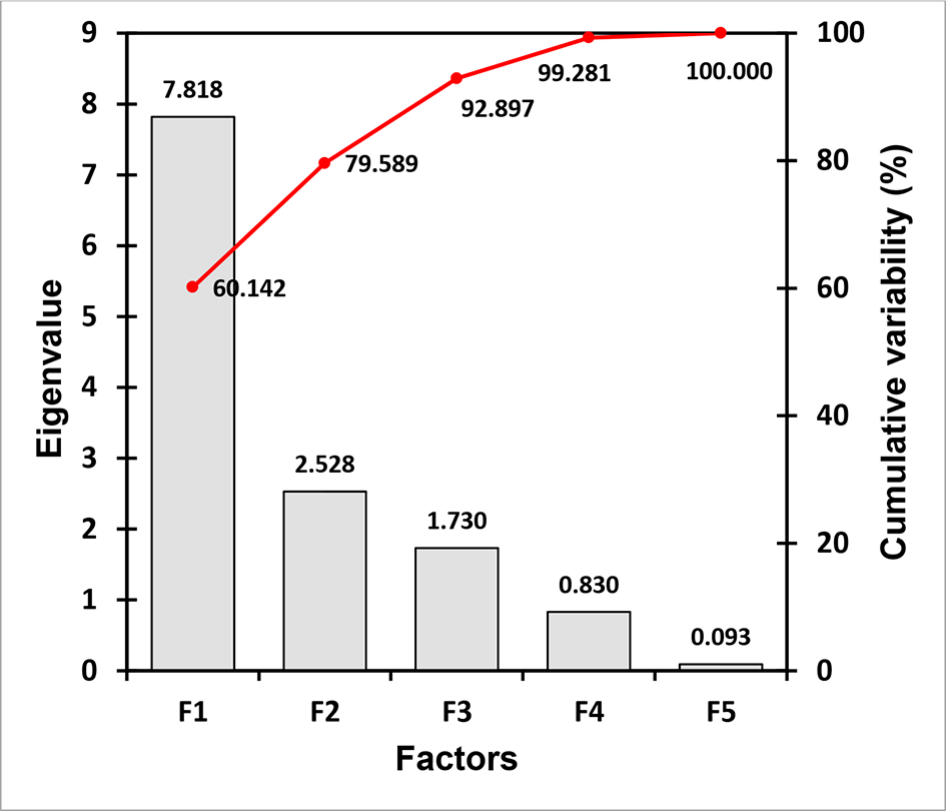
Scree plot showing the first three factors for most of the cumulative variability in data represented by the eigenvalues.

A scree plot of the eigenvalues of the correlation along the *y*-axis and he factor variables along the *x*-axis is depicted in Fig. 7. The F1 factor with n eigenvalue of 7.818 (cumulative variability: 60.14%) plus the F2 factor possessing the subsequent eigenvalue of 2.528 (cumulative variability: 79.59%) were selected for PCA in assessing the association between the heavy metal tolerance and the antibiotic resistance of the test bacteria, in addition to the F3 factor displaying an eigenvalue of 1.73, covering 92.89 % of the total variance (Fig. 7).

## Discussion

The current study demonstrated the isolation of Gram-negative as well as Gram-positive potentially pathogenic bacteria of sewage origin. Jayaseelan *et al*. [29] isolated bacteria from the dye effluent of textile mill sites (Tirupur region, Tamil Nadu, India) and characterized them biochemically to identify them as *P. aeruginosa *, *Bacillus amyloliquefaciens* and *Serattia liquefaciens*, and confirmed their identity by 16S rRNA gene sequencing and phylogenetic analysis. Abo-Amer *et al*. [30] isolated sewage wastewater bacteria, *Alcaligenes faecalis *, and identified the strain by morphological, biochemical and molecular (16S rRNA gene sequencing) characterization. Herein, the LMEM 306 strain had 100 % sequence (1482 pb) similarity to the *E. faecalis* ATCC 19433 strain (GenBank accession number: ASDA01000001), with high levels of sequence homology, having a score of ≥200, while the LMEM 305 strain showed 99 % similarity to *A. baumannii* ATCC 19606 strain (GenBank accession number: ACQB01000091), with a score of ≥200 in sequence homology, and thus, the isolated sewage wastewater bacterial strains (LMEM 306 and LMEM 305) were confirmed as *E. faecalis* LMEM 306 (GenBank accession number: MK182775) and *A. baumannii* LMEM 305 (GenBank accession number: MK182778), respectively. Such bacteria, including *E. faecalis *, are potential human pathogens [31, 32]. The *E. coli* pathogenic strains cause diverse forms of illness, such enteric and urinary tract infections, sometimes with severe complication leading to death. *P. putida *, which resides abundantly in soil and water, has been reported to cause opportunistic nosocomial infections, and possesses the capacity to cause pathogenesis [33, 34]. *A. baumannii *, which is originally an environmental bacteria, commonly found in water and soil, has currently been recognized as an emerging community and nosocomial pathogen causing life-threatening infections [35, 36].

Bacterial antibiotic resistance, which is not limited to clinical settings, has also been documented among environmental bacteria thriving in soil and water and acting as reservoirs of antibiotic resistance traits, and thus they pose a great threat to human health [37, 38]. Among the members of the family *Enterobacteriaceae *, including *Escherichia coli *, and non-lactose-fermenting bacteria such as *Pseudomonas* spp. and *Acinetobacter* spp., resistance to multiple antibiotics, including the newer ones, such as carbapenems and cephalosporins, has been reported [39]. The escalating trend for the emergence of multiple-antibiotic resistance among human pathogenic bacteria has restricted the functional capacity of antibiotics in the treatment of life-threatening infections in humans [1]. Adelowo *et al*. [40] reported the functionality of polluted urban wetlands as a reservoir of MDR bacteria: *P. putida* group (* P. plecoglossicida* and *P. guariconensis *) carrying carbapenem resistance genes. Our earlier report demonstrated multidrug resistance among clinical bacterial isolates, with *P. aeruginosa* having 8-drug resistance (Am–Ce–Cm–Ct–Cx–Nx–Pc–PT and Am–Ce–Cp–Ct–Cx–Nx–Pc–PT) phenotypes and *E. coli* having 8–10-drug resistance (Am–Ce–Cm–Cp–Cx–Mp–Nx–Pc, Am–Ce–Cp–Ct–Cx–Mp–Nx–Pc–PT and Am–Ce–Cp–Ct–Cx–Mp–Nx–Pc–PT-Tc) phenotypes [8]. Multiple antibiotic resistance has also been reported among the potentially pathogenic bacteria isolated from riverine water as well as municipal sewage water [6, 7]. In the current study, the order of resistivity found among the isolates was: *E. coli* (nine-drug resistance)>* A. baumannii* (four-drug resistance)>* P. putida */* E. faecalis* (three-drug resistance), as depicted in Table 2. Therefore, MAR Gram-negative bacteria are an ever more prevalent public health concern in our developing part of the globe. Among the *E. coli* and *Vibrio* spp. isolates from wastewater treatment plants in Eastern Cape Province (South Africa), 81 % had MAR phenotypes against ≥3 antibiotics, with ‘Am–Tc–trimethoprim–sulfamethoxazole–penicillinG–nitrofurantoin–polymyxinB’ being the common one, while the highest MAR phenotype was against 11 antibiotics (3 isolates) [17]. In the present study, the studied sewage system receives effluent from agricultural fields and mango gardens, along with domestic effluent containing excreta plus sufficient traces of antibiotics to create selection pressure for bacterial antibiotic resistance in sewage wastewater that might act as a real reservoir for antibiotic-resistant bacteria as well as antibiotic resistance genes. The occurrence of ‘antibiotic resistance genes in wastewater’ was explained earlier by Karkman *et al*. [41]. This suggests the prioritization of research on antibiotic resistance among Gram-negative bacteria for continuous public health observation activities in clinical and community settings, and among environmental bacteria as well, in order to combat MDR bacterial infection.

Earlier authors demonstrated the MAR indices to trace the nature and source of bacterial contaminants [18–20]. Frigon *et al*. [42] reported that human and animal faecal matter from wastewater plays a major role in contaminating freshwater environments with enteropathogenic bacteria. The drinking water bacterial isolates *P. aeruginosa, E. faecalis* and *E. coli* with MAR indices >0.2 demonstrated the heavy application of antibiotics in the surrounding aquatic sources [43], indicating a high risk of antibiotic contamination, which originally was reported by Tambekar [19]. As reported by Harwood *et al*. [44], *E. coli *, which is a gut bacterium of humans and animals, has been regarded as an indicator micro-organism for faecal contamination of ecological niches, including water bodies. The resistomes of *E. coli *, and other faecal bacteria, such as *E. faecalis *, from human and animal sources, disseminate resistance genes to antibiotic-sensitive environmental and subsequently human pathogenic bacteria [41, 45, 46]. Therefore, to identify the potential sources of such bacterial contaminants in local niches, MAR index-based studies are crucial [6, 7]. Alongside the Gram-positive enterococcal human pathogens, *E. faecalis* and *E. faecium* have also been recognized as faecal indicator bacteria in an aquatic environment [47]. Adefisoye and Okoh [17] demonstrated high MAR indices (0.33–0.35) among *E. coli* and *Vibrio* spp. isolates from wastewater treatment plants in Eastern Cape Province (South Africa) and suggested their (bacterial isolates) plausible origin from sources with high antibiotic contamination. Therefore, the current isolates of sewage wastewater bacteria (* A. baumannii *, *P. putida *, *E. coli* and *E. faecalis *) plausibly originated from niches with human faecal contamination and/or with high antibiotic pollution, as per the criteria explained by earlier authors [18–20].

Unsupervised anthropogenic as well as industrial action causes heavy metal accumulation in sewage where the natural bacterial populations come across the selection pressure of different heavy metals, which thus provide the basis for the emergence of heavy metal-resistant bacteria [2, 3, 48]. Abo-Amer *et al*. [30] isolated *A. faecalis* from sewage wastewater that showed multiple-heavy metal tolerance to Pb^2+^, Cd^2+^, Al^3+^, Cu^2+^, Ag^2+^ and Sn^2+^ for which the minimum inhibitory concentration (MIC) ranged from 800–1400 μg ml^−1^. Mounaouer *et al*. [49] demonstrated that among the heavy-metal-tolerant Gram-positive (* S. aureus *) and Gram-negative (* P. aeruginosa *) bacterial isolates from wastewater samples, *P. aeruginosa* was found to be more resistant to a number of heavy metals (MIC based order of toxicity: Cu >Cr> Cd>Ni> Zn>Co), while the Gram-negative isolates harboured a single plasmid. Antibiogram analysis, as demonstrated by Kaur *et al*. [50], detected the development of antibiotic resistance to Am, Cp, chloramphenicol and ceftizoxime in *Salmonella enterica* serovar Typhi alongside adaptation to cadmium after exposure to and intracellular accumulation of cadmium. As reported by Malik and Mleem [51], *Pseudomonas* spp. isolated from soil samples from agricultural field receiving irrigation including wastewater containing effluents from metal factories and domestic sewage harboured a plasmid encoding MAR to neomycin, cloxacillin and amoxicillin, and multiple heavy metal tolerance to Hg^2+^, Cd^2+^, Cr^6+^ and Cu^2+^. The emergence of MDR and carbapenem-resistant *P. putida* is a current cause of concern linked to the difficulty of treating infections [52–54], and the isolates, containing transferable R-plasmids, act as a reservoir of antibiotic resistance [55, 56]. Durve *et al*. [57], following a plasmid curing study, reported plasmid (15–26 kb)-mediated mercury resistance in a *P. aeruginosa* wastewater isolate showing resistance to multiple antibiotics. Antibiotic (Am–Cm-Ce-Cx-trimethoprim and Am-Cm-Tm-Tc-Cp-Gm) and heavy metal (Cd^2+^-Hg^2+^) co-resistance has been reported among river water bacteria as well as in clinical bacteria [6, 7]. Herein, the involvement of a ≈54 kb plasmid conferring antibiotic and heavy metal resistances has been recorded among the isolated sewage wastewater bacteria.

In environmental conditions (soil and water), heavy metals undergo a slower degradation process compared to antibiotics, and the heavy metal concentrations are thus higher than the level of antibiotics in a given niche, ensuring the persistence of selection pressure for co-resistance to heavy metals and antibiotics among bacteria [3, 58–61], wherein the expression of certain protein molecules occurs due to heavy metal stresses. Durve *et al*. [57] demonstrated the induction of a set of proteins (bands with molecular sizes of 150, 70, 50, 30 and kDa) under heavy metal (mercury, arsenic, lead and cadmium) stressors on a wastewater isolate of *P. aeruginosa *, along with the induction of the 15 kDa protein with mercury exposure, and thus, an increase in protein concentration among the bacterial isolates was established via SDS-PAGE. Chatterjee *et al*. [62] detected the expression of ≈29 kDa protein by SDS-PAGE analysis in industrial effluent isolates of bacteria with lead stress (level of resistance to lead: 6 mM), while in the absence of lead a 35 kDa protein was expressed; such heavy-metal-tolerant bacterial isolates showed resistance to single (streptomycin) and multiple (Am, Cm, rifampicin and streptomycin) antibiotics. As shown in Fig. 4, all *E. coli* strains (lanes 3–5), and *E. faecalis* ST5 (lane 6) and *P. putida* ST6 (lane 7) strains expressed two proteins (59.25 and 10.56 KDa) on exposure to Cd^+2^, while the untreated control strains (lanes 8–10), *E. coli* ST3, *E. faecalis* ST5 and *P. putida* ST6, had a smaller protein band (10.56 KDa), and both the treated and untreated control strains of *A. baumannii* ST1 only had the 10.56 KDa protein. Micro-organisms essentially require certain heavy metals for normal physiological functioning in optimum concentrations, beyond which (in situations involving repeated exposure to such chemicals) all heavy metals (including their salts) are toxic to the micro-organisms, including bacteria [63], and hence such micro-organisms, in all possible niches, express some proteins in order to persist through the heavy metal stressors.

PCA has been an important multivariate statistical approach and could be applied in justifying the association between the antibiotic resistance and heavy metal tolerance of bacteria in the given niches. Wright *et al*. [64] explained the bacterial tolerance to the heavy metals and antibiotics by conducting PCA, wherein the PC1 (principal component 1) elucidated 84.9 % of total variation, while Luo *et al*. [65] conducted PCA in explaining antibiotic resistance genes and heavy metal resistance genes among the microbial community. In the current study, PCA (Fig. 5) shows the distinctive alliances of six potential pathogenic bacteria isolated from the sewage wastewater, displaying the relationship between resistances to two environmental variables, antibiotics and heavy metals, among the bacterial isolates. A highly positive correlation between Cr^6+^ tolerance and Km resistance (Pearson’s *R﻿*=0.843) compared to Cr^6+^ and Ip (Pearson’s *R*=0.839) and Cr^6+^ and Cf (*R*=0.825) was noted, while Cu^2+^ tolerance was positively correlated to a pattern of ‘Cf >Ip> Km’ displaying Pearson’s *R* values of 0.869, 0.867 and 0.848, respectively (Table 5). Fig. 5 show 60.14 % of the total variance by F1, positively correlated to Km, Cf, Ip, Cr^6+^, Cu^2+^ and Cd^2+^ situated at quadrant I (* P. putida* ST6 strain), and to Gm, Pc, Tc, Nx and Cx in quadrant IV (* A. baumannii* ST1 and *E. faecalis* ST5 strains). The F1 was negatively correlated to Hg^2+^ and Cm in quadrants II (* E. coli* ST2, ST3 and ST4 strains) and III, respectively. The F2 factor (19.45 % of the total variance) was positively correlated to Hg^2+^ in quadrant II, and negatively correlated to Cm in quadrant III (Fig. 5); a poorly negative correlation was established between Cd and Pc (*R*=−0.056), as shown in Table 5. Two bacterial strains (Gram-positive *E. faecalis* ST5 strain and Gram-negative non-fermenting, *P. putida* ST6 strain) have been represented differently from other bacterial strains – fermenting (* E. coli* ST2, *E. coli* ST3 and *E. coli* ST4) as well as non-fermenting (* A. baumannii* ST1) – grouping with the cluster below the 4 units of the relative linkage distance according to their resistance properties (Fig. 6). The PCA performed with the resistance profiles of six sewage wastewater bacteria provided the recognition of three factors (F1, F2 and F3) with eigenvalues >1, demonstrating that the studied variables can plausibly be grouped into three factors explicating 92.89 % of the entire variance (Fig. 7). The rest of the factors (F4 and F5) account for a very little of the variability, and were thus inconsequential; similar events had been expressed by earlier authors [28, 66].

Thus, the Gram-negative (* E. coli *, *n*=3; *A. baumannii *, *n*=1; and *P. putida *) and Gram-positive (* E. faecalis *; *n*=1) clinically relevant bacteria that were isolated from the wastewater of a sewage system receiving various kinds of polluted effluents had high MAR indices, and the test bacteria had a single plasmid (≈54 kb) conferring multiple antibiotic resistances linked to the tolerance of a number of heavy metals (Hg^+2^, Cd^+2^, Cr^+6^ and Cu^+2^). The sewage wastewater might act as a reservoir of multiple-antibiotic-resistant bacteria and the R-plasmid contained in them, potentially causing the dissemination of MAR and heavy metal tolerance genes, impacting the environment as well as human health in this part of the globe. Hence, vigilance and regular monitoring of the R-plasmid in environmental settings are mandatory to minimize the global emergence and spread of bacterial multiple-antibiotic resistance.
